# Supplementary figures and images for: Evidence of disorientation towards immunization on online social media after contrasting political communication on vaccines. Results from an analysis of Twitter data in Italy
Source: PLoS One. 2021 Jul 9;16(7):e0253569. doi: 10.1371/journal.pone.0253569 (PMC8270452; doi:10.1371/journal.pone.0253569)

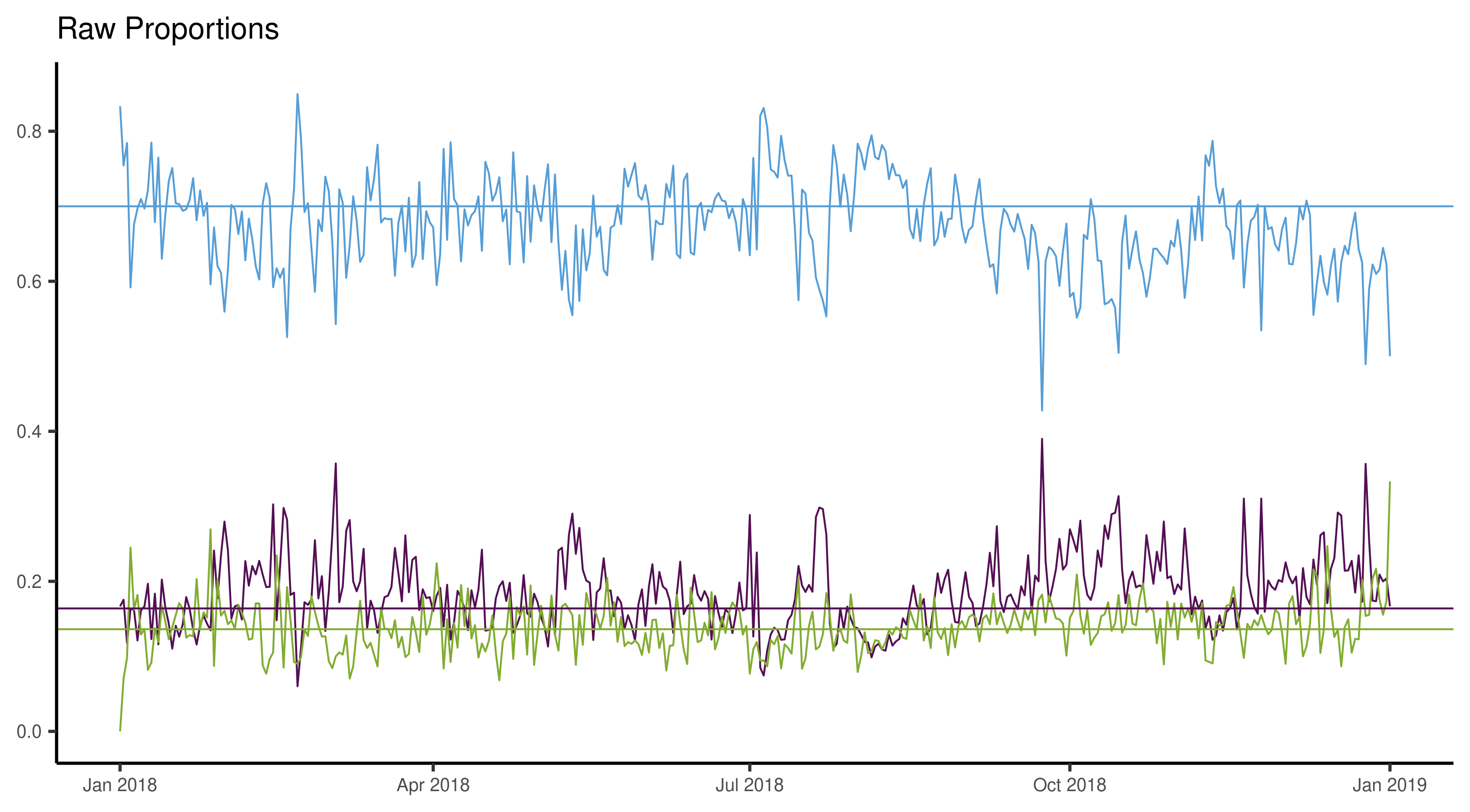

Supplement: S1 Fig — (TIF) [file pone.0253569.s001.tif]
