# Supplementary material for: Evidence of disorientation towards immunization on online social media after contrasting political communication on vaccines. Results from an analysis of Twitter data in Italy
Source: PLoS One. 2021 Jul 9;16(7):e0253569. doi: 10.1371/journal.pone.0253569 (PMC8270452; doi:10.1371/journal.pone.0253569)
Supplement: S1 File — (DOCX) [file pone.0253569.s002.docx]

***Supporting information and Online appendix***

1. ***Results of automatic classification analyses.***

We report here the classification results of the remaining four algorithms discussed in the Material and Methods.

**Random Forest**

|  | precision | recall | **f1-score** | support |
| --- | --- | --- | --- | --- |
| Favorable | 0.50 | 0.27 | **0.35** | 785 |
| Contrary | 0.37 | 0.04 | **0.07** | 318 |
| Undecided | 0.33 | 0.01 | **0.01** | 299 |
| Out of Context | 0.56 | 0.89 | **0.67** | 1460 |
| accuracy |  |  | **0.53** | 2862 |
| macro avg | 0.44 | 0.30 | **0.28** | 2862 |
| Weighted avg | 0.49 | 0.53 | **0.45** | 2862 |

***S1 Table: Report of the result for classification with Random Forest***

**Naive Bayes**

|  | precision | recall | **f1-score** | support |
| --- | --- | --- | --- | --- |
| Favorable | 0.45 | 0.46 | **0.46** | 785 |
| Contrary | 0.27 | 0.06 | **0.10** | 318 |
| Undecided | 0.30 | 0.05 | **0.09** | 299 |
| Out of Context | 0.60 | 0.80 | **0.69** | 1460 |
| accuracy |  |  | **0.55** | 2862 |
| macro avg | 0.41 | 0.34 | **0.33** | 2862 |
| Weighted avg | 0.48 | 0.55 | **0.50** | 2883 |

***S2 Table: Report of the result for classification with Naive Bayes***

**KNN**

|  | precision | recall | **f1-score** | support |
| --- | --- | --- | --- | --- |
| Favorable | 0.41 | 0.37 | **0.40** | 785 |
| Contrary | 0.22 | 0.10 | **0.14** | 318 |
| Undecided | 0.14 | 0.04 | **0.07** | 299 |
| Out of Context | 0.28 | 0.75 | **0.66** | 1460 |
| accuracy |  |  | **0.51** | 2862 |
| macro avg | 0.34 | 0.32 | **0.31** | 2862 |
| Weighted avg | 0.45 | 0.51 | **0.46** | 2862 |

***S3 Table: Report of the result for classification with KNN***

**Classification Tree**

|  | precision | recall | **f1-score** | support |
| --- | --- | --- | --- | --- |
| Favorable | 0.49 | 0.26 | **0.34** | 785 |
| Contrary | 0.16 | 0.02 | **0.04** | 318 |
| Undecided | 0.23 | 0.02 | **0.04** | 299 |
| Out of Context | 0.51 | 0.88 | **0.67** | 1460 |
| accuracy |  |  | **0.52** | 2862 |
| macro avg | 0.35 | 0.29 | **0.27** | 2862 |
| Weighted avg | 0.45 | 0.52 | **0.44** | 2862 |

***S4 Table: Report of the result for classification with Classification Tree***

**Report of Cross-Annotator Evaluation**

|  | precision | recall | **f1-score** | support |
| --- | --- | --- | --- | --- |
| Favorable | 0.65 | 0.62 | **0.63** | 388 |
| Contrary | 0.46 | 0.38 | **0.42** | 154 |
| Undecided | 0.23 | 0.26 | **0.24** | 120 |
| Out of Context | 0.73 | 0.76 | **0.74** | 689 |
| accuracy |  |  | **0.63** | 1351 |
| macro avg | 0.52 | 0.50 | **0.51** | 1351 |
| Weighted avg | 0.63 | 0.63 | **0.63** | 1351 |

***S5 Table: Report of the result for manual human agreement annotation***

1. ***The keywords adopted in the analysis***

| **Context** | **Italian keyword (English translation)** |
| --- | --- |
| Vaccination topic | “copertura vaccinale” (vaccination coverage); “vaccini”, “vaccino” (vaccine(s)); “vaccinazione” (Vaccination); “iovaccino” (Ivaccine), “comilva”; “corvelva”; “thimerosal”, “esami prevaccinali” (prevaccination exams); “lobby vaccini”; “vaxxed”; “trivalente” (trivalent); “esavalente” (hexavalent); “obbligo vaccinale” (mandatory vaccines); “varicella party” (chickenpox); “autismo” (autism); “lobby vaccini” (vaccine’s lobby); |
| Vaccine-preventable diseases | “meningite” (meningitis), “morbillo” (measles); “rosolia” (rubella); “parotite” (mumps); “pertosse” (whooping cough); “poliomelite” (polio); “varicella” (chickenpox); “MPR” (italian acronym for measles, mumps, rubella); “HPV”, |
| Hashtags | #novaccino (“no vaccine”); #iovaccino (“I vaccinate”); #libertadiscelta (“freedom of choice”); “#vaxxed” |

***S****6 Table: Keywords adopted to retrieve the Tweets.*

***Examples of tweets by category***

- **Favorable:** "Chi non vaccina se stesso e i propri figli se questi si ammalano deve essere sanzionato penalmente. Assumetevi le vostre responsabilitá se volete giocare sulla pelle degli altri. #provax"
- **Contrary:** “GiuliaGrilloM5S DENUNCIATA la #Lorenzin: ha nascosto documenti che svelano i DANNI dei # VACCINI !!! Strano…”
- **Undecided:** “Non sono contro i vaccini a prescindere, ma visto che li dobbiamo iniettare nel corpo dei nostri figli, mi sembra un nostro diritto sapere cosa c’è esattamente nel vaccino e quali potrebbero essere...”
- **Out-of-context: “**Vaccino antitumore, potrebbe essere disponibile entro un anno: Elimina il cancro senza chemioterapia”

***Tweets Annotation***

*The file “annotated_tweets_ids” represents the manually annotated training set used for the analysis.
We have reported for each tweet ID the label (sentiment) assigned by the annotator according to the following rules:*

- ***P***: the tweet is favorable
- **N**: the tweet is contrary
- **H**: the tweet is undecided
- **Z**: the tweet is out-of-context
