# Supplementary material for: Evidence of disorientation towards immunization on online social media after contrasting political communication on vaccines. Results from an analysis of Twitter data in Italy
Source: PLoS One. 2021 Jul 9;16(7):e0253569. doi: 10.1371/journal.pone.0253569 (PMC8270452; doi:10.1371/journal.pone.0253569)
Supplement: Attachment — Submitted filename: review.pdf [file pone.0253569.s004.pdf]

Review for manuscript "Evidence of distrust and disorientation towards immunization on online social media after contrasting political communication on vaccines. Results from an analysis of Twitter data in Italy."

In this work the authors are analyzing vaccination-related data retrieved from Twitter from 2018 in Italian language and put into the political context during this time. A subset of the data was annotated into 4 categories, those being "favorable", "contrary", "undecided" and "out of context" and a Machine Learning classifier was trained on this data. Predicted data by this classifier was subsequently analyzed, particularly with respect to the absolute counts in each category and their temporal trends. Overall, most tweets were categorized "out of context". Among the relevant category, most tweets were determined to be "favorable" and the rest was subdivided into the categories "contrary" and "undecided". Polynomial fitting was applied to the sentiment trends showing a decline of the "favorable" group towards the end of the year, as well as a slight increase in "contrary" and especially "undecided". The authors then discuss a possible relation between the change of the government to the way vaccination is discussed on Twitter. One of the general conclusions is an increase in "disorientation" due to the ambiguous announcements made by the new government.

The work proposed is interesting and focuses on a relevant topic. However, there is a mismatch between the presented results and the discussion section. The conclusion of there being a direct link between the change of government and the decline in vaccination sentiment and increase in "disorientation" needs to be discussed more clearly. There are several parts of the paper which are unclear and need to be rewritten. I therefore suggest a major revision of this manuscript before publication.

Note that the comments are not given in a specific order. Also, I have not corrected any grammatical mistakes.

Methods

- (minor) The authors mention a total of 4 classes ("favorable", "contrary", "undecided" and "out of context"). It is unclear whether the algorithm was trained on 4 classes or only on 3 classes. If the "out of context" class was simply removed then it means that the predicted data will come from a different underlying distribution than the training data (which could be problematic and should at least be mentioned).

- (minor) Precision, recall and F1 were given for the classifiers. It would be helpful to know the F1 scores for each subclass. Furthermore, it should be mentioned whether these scores are micro or macro averages.
- (minor) Lines 139-144 need better explanation and phrasing. What test was used to determine the degree of freedom for the smoothing? What kernel smoothing procedure?
- (minor) It is not mentioned whether the data was collected through the Twitter API (if so, which endpoint was used?) or via the website. If data was collected via the website it should be written (potentially in the discussion) that the search is not exhaustive and the returned data is filtered by Twitter in terms of relevance/trendingness, which might bias the analysis.
- (minor) It would be very much appreciated if the tweet IDs were published together with the code. This would allow other researchers to reproduce these results. Additionally, given the effort in collecting the annotation data, releasing this data would increase the impact of the work significantly.

Results

- (minor) Figure 1 lacks y-axis labels and legend for the color bar
- (major) It is unclear how the "disorientation" was measured and how it relates to the observed signal. If disorientation is simply a result of the up-and-down trend then one could e.g. plot the variance of the signal over time and see if it increases "sharply" when the government changed. The term "disorientation" is only mentioned in the abstract, title and the beginning of the results section but not in the discussion.

Discussion

- (minor) *"After removing noise, the population appeared to be mostly composed by “serial- twitterers” i.e., people tweeting about everything “on top”, including also vaccines, regardless of their awareness of the topic."* (Lines 234-236)
  What do the authors mean by "serial-twitterers", a group of normal twitter users which also tweet about other things than vaccines? If so, how do the authors know since not all tweets from the timelines of these users were collected? It is also not clear what the term "on top" means in this context. I would recommend to not use the term "serial twitterer" and instead describe this group in another way. Also authors should provide some sort of quantitative reasoning/support for how they allocated users to this group.
- (major) Lines 247-258 discuss how the MMR vaccine coverage relates to the sentiment observed. This should be either moved to the results section or (as the authors state) if not part of the main message of this manuscript it should not be discussed at all. The question of correlation between sentiment and vaccine coverage is an important one, but should be analyzed in more detail and by contrasting e.g. with data from opinion polls before a clear link can be made between Twitter sentiment and vaccination coverage. There is also important literature on this topic which would need to be included in this

type of analysis.
"As for the limitations of this work, the main critical point lies in the general relevance of opinion-based information from OSM for predicting trends of vaccine uptake." (Lines 295-296)
The authors mention this as the main limitation of this study. However, as mentioned above vaccine uptake was not properly studied. Therefore, this caveat doesn't apply here.

- (minor) *"A key problem is the appropriate modulation of the "language style" to be used by public health communication on online social media."* (Lines 280-281)
  Since no analysis on language style was performed this should be either left out or rephrased. If kept, authors should include appropriate literature on this topic.
- (minor) *"We plan to deep(en) this in future research [...]",* (Line 281)
  The mentioned research sounds important, but a bit misplaced in the middle of the discussion of the results. Future research should be summarized in a general sense (what is the future research needed to be done by the community as a whole?) at the end and discussed together with caveats.
- (major) *"A specific search was therefore carried out over the set of retained tweets by further keywords specifically targeting this situation [...]"* (Lines 120-121)
  It is unclear which fields of the tweets were searched (user description, text, etc.)? It is also unclear how (if a tweet matched any of the provided keywords) this would directly identify said tweeter as a parent with children in the age of childhood immunization. Later in the discussion it is mentioned that the number of tweets matching the criteria was really small (line 244), therefore it was not analysed further. Although I appreciate the inclusion of negative results, it would be better to move most of it to the results section. Furthermore, as this approach was not successful what was the reason for this? Have the authors tried to expand the search to other keywords? Was the total body of tweets not large enough? The discussion should also involve issues related to identifying demographic subgroups by simple keyword matching (which is obviously problematic).
- (major) *"In relation to the growing literature on sentiment analyses and vaccines this is, to the best of our knowledge, the first work on the subject documenting a clear medium-term distrust effect towards immunization arising from persistently ambiguous positions at the highest political level."* (lines 291-293)
  "Resulting from" is a strong statement, implying direct causation just by observing minor correlations (R2 values are relatively low). This seems to be the main hypothesis of this work but it is not properly discussed. One possible way to discuss causality would be using the Bradford Hill criteria (strength, consistency, temporality, etc.) Some of these criteria might match better, others worse.
- (major) Lines 303-309 are contrasting Twitter to Facebook data and the observation of echo chambers. No Facebook data was analyzed in this study, hence I don't see the need to contrast the collected data with Facebook data. Furthermore, no analysis was conducted with regards to the effects of echo chambers. It is important to address the issues of Twitter data, but it should be limited with respect to the analysis & conclusions

in the manuscript.
